# Resolution of Anastomotic Ulceration and Profound Bleeding by Endoscopic Cauterization and Oral Budesonide

**DOI:** 10.1097/PG9.0000000000000328

**Published:** 2023-06-16

**Authors:** Dong Xi, Melanie Hakar, Jessica Davis, Henry Lin

**Affiliations:** From the * Pediatric Gastroenterology, University of Tennessee Health Science Center, Memphis, TN; † Pathology and Laboratory Medicine, Oregon Health & Science University, Portland, OR; ‡ Pediatric Gastroenterology, Oregon Health & Science University, Portland, OR.

**Keywords:** Anastomotic ulceration, argon plasma cauterization, budesonide, short gut syndrome

## Abstract

Anastomotic ulceration is a known complication of short gut syndrome, which can be complicated by concurrent iron deficiency and chronic bleeding. Diagnosis is confirmed through endoscopy, typically performed for the investigation of severe anemia. Inflammatory colitis in short gut syndrome has been previously reported; therefore, it is important to treat both ulceration and inflammation. Currently, no effective therapies are available. Herein, we describe the case of a child with short gut syndrome who subsequently developed anastomotic ulcers with recurrent severe bleeding and was successfully treated with endoscopic argon plasma cauterization for circumferential ulcerations, followed by a prolonged course of oral enteric budesonide. This intervention could be a potential and beneficial nonsurgical treatment for anastomotic ulceration.

## INTRODUCTION

Anastomotic ulceration (AU) is a known complication of short gut syndrome and typically occurs when a portion of the small bowel is resected (1), especially when the ileocecal valve is resected too. It occurs in the pediatric population with a reported incidence rate of 7% in a cohort of 114 children with short gut syndrome and can result in iron deficiency and chronic bleeding (2). Currently, no effective therapies have been established. Medications, surgical resection, and revision of the anastomosis can be attempted, although they are not always successful. Oral budesonide has been utilized for gastrointestinal inflammation (3), suggesting a possible benefit for patients with AU.

Herein, we described a case of a child with short gut syndrome who subsequently developed AU that was complicated by severe, recurrent gastrointestinal bleeding. The AU was successfully managed by endoscopic argon plasma cauterization (APC) for circumferential ulcerations, followed by a prolonged course of oral enteric budesonide. The success of this intervention could be a promising and beneficial treatment for children with AU.

## CASE REPORT

A 10-year-old patient presented with recurrent anemia. The patient was born at a gestational age of 34 weeks and was hospitalized in the neonatal intensive care unit for 4 months due to gastroschisis and intestinal atresia. The patient underwent multiple bowel resections, including removal of the terminal ileum, resulting in short gut syndrome. Anastomosis was performed via interrupted technique with a silk suture, during which time a gastrostomy tube was placed and the patient was total parental nutrition dependent. The patient underwent a subsequent serial transverse enteroplasty procedure at 2 years old. Enteral feeding was advanced slowly and the patient was weaned off total parental nutrition by age 3 years. The patient’s oral feeding skills improved gradually and tube feeding was discontinued with permanent gastrostomy tube removal at 4 years old.

The patient developed anemia by the age of 5 years with hemoglobin and ferritin levels of 6.4 g/dL and 3 ng/mL, respectively, requiring blood transfusions. The patient was found to have hemoccult-positive stools and underwent esophagogastroduodenoscopy (EGD), which revealed gastric ulcers. The biopsy was negative for *Helicobacter pylori* infection, and the colonoscopy was unremarkable. Omeprazole to manage the ulcers and iron supplements were initiated. Follow-up iron and ferritin levels at 3 months did not demonstrate significant improvement. Nausea and vomiting became associated with the iron supplements, so they were switched to intravenous iron infusions weekly for 4 doses. Afterward, the patient developed 2 episodes of pallor with significant fatigue and shortness of breath. Upon evaluation, the patient had hemoglobin levels of 4.4 and 6.8 g/dL, which prompted emergency transfusions. Iron infusions were restarted monthly for 5 years and eventually ferritin levels normalized.

**FIGURE 1.**
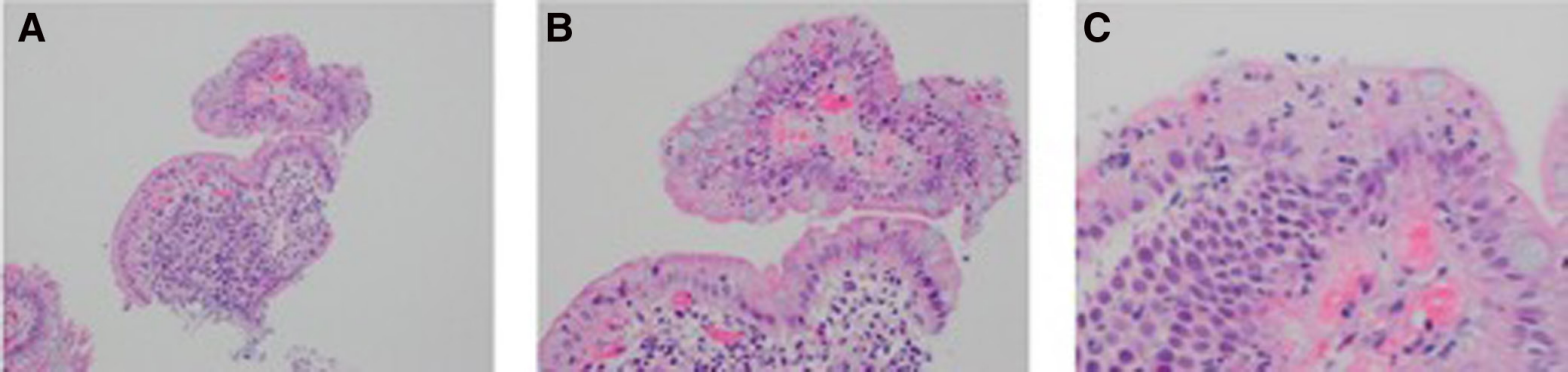
Histological images showing chronic epithelial inflammation of anastomotic biopsies at different magnifications (A–C).

**FIGURE 2.**
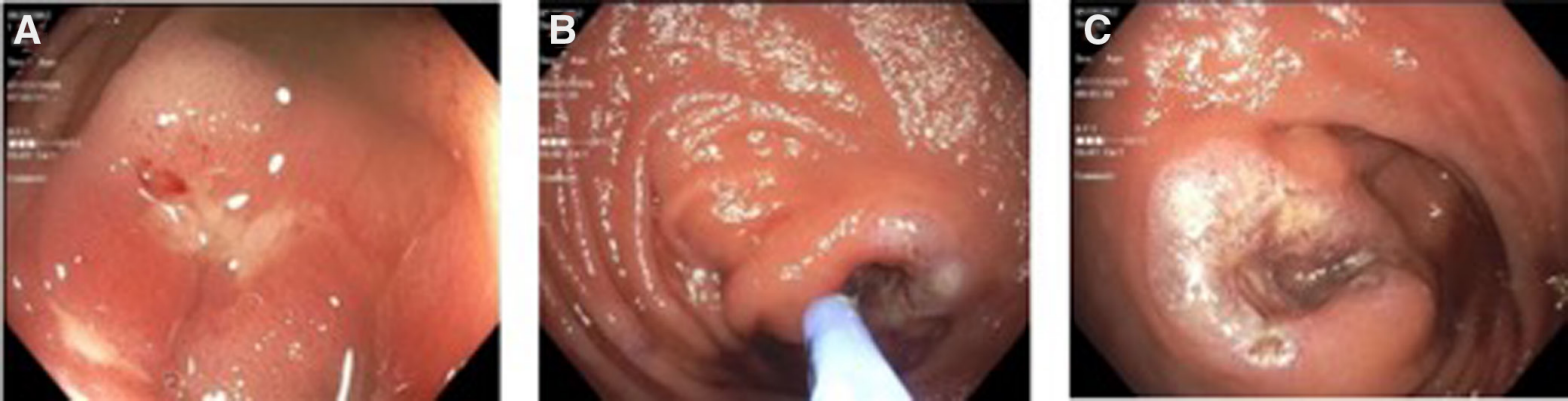
Endoscopic images showing severe ulcerations with stigmata at the ileocolonic anastomosis (A), which was successfully cauterized by APC (B and C). APC = argon plasma cauterization.

**FIGURE 3.**
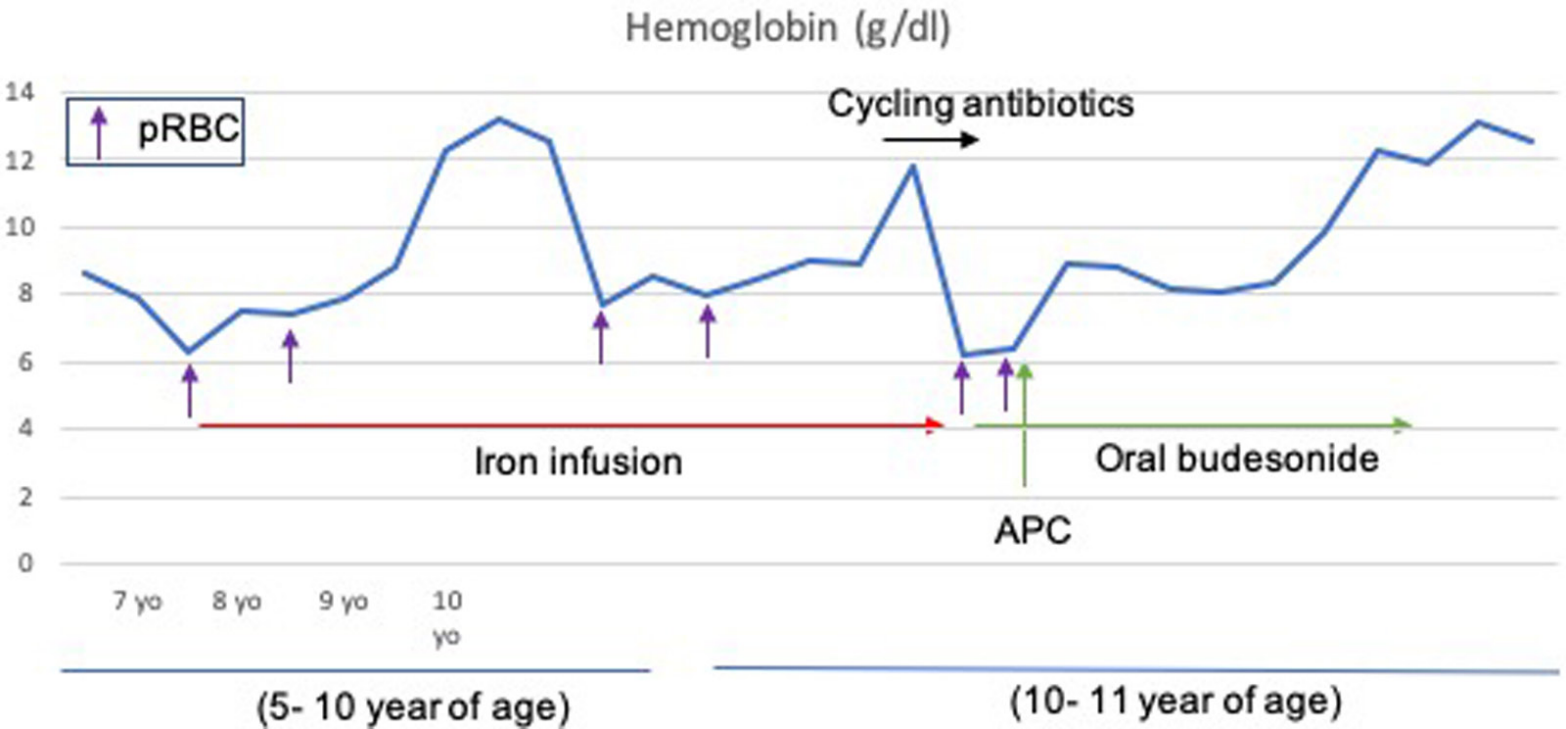
Graph showing the timeline of treatments the patient received and the levels of hemoglobin at different ages before and after endoscopic APC and oral budesonide. APC = argon plasma cauterization.

At a clinic visit at 10 years old, a care plan was discussed with the parents, which included empiric cycling antibiotics for small intestinal bacterial overgrowth (4,5) and an attempt to wean the patient off the iron infusion; the family agreed. Two months after the iron infusion was temporarily paused, the patient presented to the emergency room again with severe pallor and fatigue. A hemoglobin level of 6.2 g/dL was found, which did not improve with blood transfusion. The patient underwent urgent EGD and colonoscopy, which revealed normal EGD and resolution of previous gastric ulcers but multiple circumferential AUs with stigmata that were cauterized without complications via APC (Figs. 1 and 2). Oral enteric-coated budesonide (Entocort EC) at 9 mg daily was also started with continued oral iron supplementation for 3 months until iron and ferritin levels normalized. The patient was closely monitored, and hemoglobin improved and then normalized. It has remained within the normal range for the next 6 months (Fig. 3), and budesonide was discontinued after 9-month treatment. Repeated hemoglobins are noted to be stable.

## DISCUSSION

AU can occur at any time after the initial resection of the small bowel (6,7). Its etiology remains unclear, although it could include exposure of the ileal mucosa to the colonic microenvironment, which disturbs mucosal integrity, or relative ischemia secondary to fibrosis at the anastomotic site (8). A diagnosis of AU is made through colonoscopy or capsule endoscopy (9), which is typically performed to investigate iron deficiency/anemia (5). When inflammatory colitis at the ulceration region has been reported, the resolution of chronic bleeding may require treatment for both ulceration and inflammation.

In this case report, our patient developed substantial anemia at 5 years of age, which is consistent with the reported age of onset for AU (ie, 3–13 years postresection of the small bowel) (10). There is currently no effective regimen for preventing AU; therefore, management is focused on therapeutic interventions after diagnosis. A wide variety of therapies have been tried, including surgical resection and revision of the anastomosis, which has not always been successful. Resection/revision also has potential complications of anastomotic leakage, recurrent bleeding, and malnutrition. A nonsurgical approach should, therefore, be considered, except in cases of life-threatening bleeds. The combined therapeutic regimen, as we have demonstrated, proved to be effective and sustainable in preventing the recurrence of bleeding from AU. It could be a potential treatment option.

Complications, including late marginal ulceration, were probably related to short gut syndrome, ileocecal valve removal, and anastomosis. Ulcer locations indicated that a change in the intestinal environment toxic to both ileal and colonic mucosa caused mucosal damage (11). The development of short bowel inflammation was also attributed to the fermentation of nutrients from malabsorbed carbohydrates, which improved with oral budesonide in our patient. Budesonide is a new-generation corticosteroid that allows local, selective treatment of the gastrointestinal tract, exerting potent anti-inflammatory effects via high affinity to the intracellular glucocorticoid receptor.

In summary, the management of AU should be focused on treating both the ulceration and the underlying noninfectious inflammation. We presented this case to highlight the potential of using endoscopic APC with oral budesonide as a treatment for short gut syndrome with AU and profound bleeding.

## ACKNOWLEDGMENTS

Scientific and technical editing was provided by Andrew J. Gienapp (Children’s Foundation Research Institute at Le Bonheur Children’s Hospital, Memphis, TN).

Informed patient consent was obtained from legal guardians for publication of the case details.
